# La teneur en iode du sel de cuisine consommé à Lubumbashi et le statut iode des personnes vulnérables: cas de femmes enceintes de milieux défavorisés

**DOI:** 10.11604/pamj.2016.23.129.7645

**Published:** 2016-03-25

**Authors:** Bienvenue Ilunga Banza, Jean Baptiste Simbi Lumbu, Philippe Donnen, Eugène Kabange Twite, Daniel Mikobi Kwete, Costa Mwadianvita Kazadi, Jean Okolonken Ozoza, Laurence Habimana, Prosper Muenze Kayamba Kalenga, Annie Robert

**Affiliations:** 1 Département de Chimie, Faculté des Sciences/Université de Lubumbashi, Lubumbashi, RD Congo; 2 Ecole de Santé Publique, Université Libre de Bruxelles, Bruxelles, Belgique; 3 Département des Sciences Biomédicales et Ecole de Santé Publique, Faculté de Médecine UNILU, Lubumbashi, RD Congo; 4 Département de Sociologie et Anthropologie, Faculté des Sciences Sociales UNILU, Lubumbashi, RD Congo

**Keywords:** Sel de cuisine, disponibilité en iode, statut iodé, femmes enceintes, Lubumbashi, Cooking salt, iodine availability, iodine status, pregnant Women, Lubumbashi

## Abstract

**Introduction:**

La consommation du sel faiblement iodé peut engendrer des troubles divers liés à la carence iodée Ce travail a pour objectif d’évaluer la teneur en iode du sel consommé à Lubumbashi et de déterminer le statut iodé des femmes enceintes, cible privilégiée de la carence iodée.

**Méthodes:**

Une étude transversale descriptive a été consacrée à une analyse iodométrique d'iode dans 739 échantillons de sel collectés dans les ménages et marchés de Lubumbashi en 2014. Précédemment, l'iode urinaire a été déterminé par la technique de minéralisation au persulfate d'ammonium chez 225 femmes enceintes reçues en consultation du 15 mars 2009 au 25 avril 2011.

**Résultats:**

Notre enquête a révélé 47,5% des échantillons de sels de cuisine adéquatement iodés (15 à 40 ppm), 36,9% d’échantillons faiblement iodés, 7,4% d’échantillons trop riches en iode et 8,1% des échantillons non iodés. La disponibilité en iode du sel de cuisine analysé était globalement de 54,9%, se trouvant nettement en dessous des normes OMS (90%). En mesurant l'iode urinaire chez la femme enceinte, la carence iodée (iode urinaire <150 µg/l) a été observée dans une proportion de 52%.

**Conclusion:**

La faible disponibilité en iode du sel consommé à Lubumbashi pourrait être responsable d'une grande proportion de la carence iodée observée chez la femme enceinte, ce qui expose celle-ci aux risques majeurs des troubles dus à la carence en iode.

## Abstract (en)

**Introduction:**

Consumption of low iodine salt can cause different types of disorders associated with iodine deficiency. This study aims to determine iodine content in table salt consumed in Lubumbashi and iodine status of pregnant women who are the main target of iodine deficiency.

**Methods:**

aAdescriptive cross-sectional study was devoted to an iodometric iodine analysis of 739 salt samples collected from the households and the markets of Lubumbashi in 2014. Previously, urinary iodine concentrations were determined in 225 pregnant women received for consultation from 15 March 2009 to 25 April 2011 by mineralization technique using ammonium persulphate.

**Results:**

Our survey found that 47.5% of the cooking salt samples were adequately iodized (from 15 to 40 ppm), 36,9% of the samples had low iodine levels, 7,4% of the samples had too much iodine and 8,1% of the samples were not iodized. Iodine concentration in the analyzed cooking salt reached an overall average of 54,9%, being clearly below the WHO Standards (90%). By measuring urinary iodine concentration of pregnant women, iodine deficiency (urinary iodine <150 µg/l) was observed in 52%.

**Conclusion:**

The low availability of iodine from consumed salt in Lubumbashi could be responsible for a large proportion of the observed iodine deficiency in pregnant women, exposing them to the major risks for disorders associated with iodine deficiency.

## Introduction

L'iode est un micronutriment indispensable à l'organisme humain. C'est une substance fondamentale pour l’élaboration des hormones thyroïdiennes et il suffit, pour cela, de disposer de petites quantités d'iode, 100 à 200 µg par jour. Son apport est essentiellement alimentaire [1]. Le déficit d'apport alimentaire quotidien en iode est responsable d'une série d'anomalies qualifiées de «troubles dus à la carence en iode» (TDCI). C'est notamment l'hypothyroïdie, le goitre, l'arriération mentale (crétinisme), l'anémie, les avortements spontanés, la mortalité infantile et la diminution de la fertilité [2]. En 1990, 30% de la population mondiale (1,5 milliard d'individus) étaient exposés à un risque de carence en iode [1]. C'est ainsi que l'OMS a recommandé l'iodation universelle du sel comme stratégie de prévention et de contrôle des TDCI. Le nombre de pays ayant une carence en iode comme un problème national de santé publique, a diminué passant de 110 pays en 1993 à 47 pays en 2007 [3]. De nombreux facteurs influencent le choix d'une teneur en iode appropriée pour une population donnée. Il s'agit notamment de la consommation moyenne de sel par jour et par personne, du degré de carence en iode, de la perte de l'iode au cours de la chaîne de distribution et de la durée d'entreposage du sel [4]. L'iodation universelle du sel varie d'un pays à un autre. Les taux d'iodation du sel sont de 40 à 80 ppm en Tanzanie [5], 30 à 50 ppm en Côte d'Ivoire [6], 50 à 100 ppm au Cameroun dans les entreprises productrices de sel [4].

Au regard de cette situation, la République Démocratique du Congo (RDC) dont tout le sel d'alimentation humaine est importé a, comme la plupart des autres pays, adhéré au programme de prévention et de contrôle des TDCI par adoption de la stratégie universelle d'iodation du sel de qualité alimentaire. Cet engagement s'est traduit en 1993 par la signature de l'Arrêté interministériel n^°^ 001 du 28 octobre 1993 portant réglementation de la production, du contrôle de qualité et de la commercialisation du sel iodé. Des mesures d'application de cette réglementation nationale ont été mises en vigueur dès l'année 1994, notamment l'interdiction de l'importation du sel non iodé et le contrôle de son iodation à différents niveaux de son circuit de distribution. Selon les textes légaux utilisés par l'Office Congolais de Contrôle (OCC), cette teneur était comprise entre 30 et 100 ppm à la production [7], au début de l'adoption du programme universel d'iodation du sel. Actuellement, la RDC exige une teneur en iode de 40 ppm à la production du sel pour que celui-ci parvienne aux consommateurs avec une teneur d'iode dans le sel comprise entre 15 et 40 ppm [8]. Pour la promotion de cette stratégie, des mesures incitatives ont été prises spécialement l'allégement des charges fiscales pour l'importation du sel iodé [9]. Une enquête réalisée en 2007 sur toute l’étendue de la RD Congo auprès de 3240 enfants en âge scolaire avait montré une disponibilité de 97,5% de sel iodé dans les ménages. La prévalence de goitre était d'environ 2,8% mais aucun cas de crétinisme n'avait été observé [10]. Au Katanga, la disponibilité de sel iodé dans les ménages était évaluée à 92,4% dans l'enquête nationale menée en 2007 [10] mais aujourd'hui, il n'existe à ce sujet aucune information disponible. La présente étude a pour objectif d’évaluer la teneur en iode du sel de cuisine consommé à Lubumbashi et de déterminer le statut iodé des femmes enceintes, cible privilégiée de la carence iodée.

## Méthodes

L'enquête porte sur une étude transversale descriptive effectuée dans quelques ménages et marchés de Lubumbashi. La première phase du travail a été réalisée du 15 mars 2009 au 25 avril 2011 chez 375 femmes parmi lesquelles se retrouvent 225 femmes enceintes reçues en consultation dans trois centres hospitaliers: les cliniques universitaires de Lubumbashi en milieu urbain avec une population de niveau de vie aisé, le centre de santé Bongonga en milieu semi-urbain avec une population de niveau de vie modeste et l'hôpital général de référence de la Katuba en milieu rural avec une population de niveau de vie précaire. Le recueil des données sur les ménages des femmes lors de l'interview de celles-ci. Les femmes interviewées devaient répondre selon la séquence des questions et avaient la possibilité à la fin de l'entretien de discuter et de poser des questions à l'enquêteur. Elles ont été invitées à fournir le lendemain dans un tube cylindrique en polyéthylène propre, sec et fermé environ 20 grammes (une à deux cuillères à soupes) de sel de cuisine prélevé sur le volume de sel stocké au domicile au moment de l'enquête. La deuxième phase de l’étude a porté sur sept marchés de Lubumbashi où l’équipe d'enquêteurs a procédé en 2014 à l'identification des vendeurs détaillants et des fournisseurs du sel. Le dosage de l'iode dans le sel a été réalisé par la méthode de titration volumétrique par iodométrie et l'iode urinaire a été déterminé chez 225 femmes enceintes par la technique de minéralisation au persulfate d'ammonium, basée sur la réaction de Sandell Kolthoff [11]. Les données collectées ont été analysées à l'aide des logiciels Excel XLSTAT et SPSS.

## Résultats

### Teneur en iode du sel de cuisine consommé à Lubumbashi

**Figure 1:**
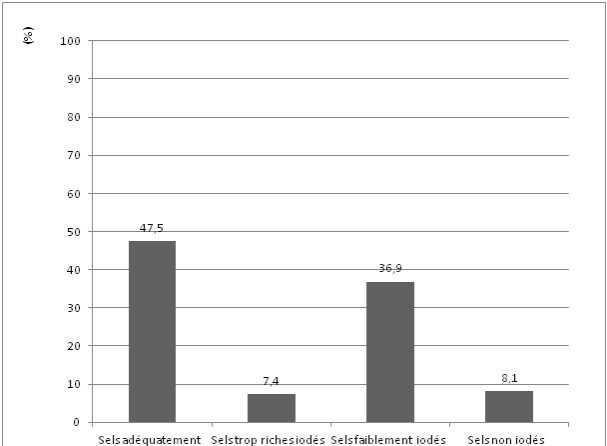
Proportion de différentes catégories des teneurs en iode des sels de cuisine prélevés dans les ménages et les marchés de Lubumbashi (n = 739)

Notre étude montre que 47,5% des échantillons de sels de cuisine consommé à Lubumbashi sont correctement iodés conformément à la norme recommandée par l'OMS (15 à 40 ppm), 36,9% des échantillons de sels de cuisine ont une teneur en iode inférieure au seuil minimal recommandé (< 15 ppm), 7,4% présentent une teneur en iode qui dépasse la limite supérieure recommandée (40 ppm) et 8,1% des échantillons de sels de cuisine ne sont pas iodés (0 ppm) (Figure 1).

### Le statut iodé des femmes enceintes à Lubumbashi

En analysant les concentrations d'iode urinaire chez 225 femmes enceintes, il a été noté que la médiane d'iode urinaire était de 138 µg/l, se situant en dessous du seuil minimal de 150 µg/l recommandé par l'OMS. La carence iodée (iode urinaire < 150 µg/l) a été observée dans une proportion de 52% (tableau III), autrement dit une femme enceinte sur deux présente une faible concentration d'iode. Par ailleurs’> témoin d'une carence en iode. Par ailleurs nos résultats ont montré qu'au cours de la grossesse, la carence en iode était plus fréquente au troisième trimestre (68%) et au deuxième trimestre (56%) qu'au premier trimestre (31%). Moins de 20% de femmes enceintes avaient un statut iodé normal (iode urinaire entre 150 et 249 µg/l). Paradoxalement 23% de femmes enceintes étaient confrontées à une surcharge en iode (250-499 µg/l) et 7% à un excès d'iode (>500 µg/l) (Tableau 1). L'examen du statut iodé chez les femmes suivant le milieu de recrutement a montré une plus grande proportion des femmes avec une carence iodée en zone rurale (6%) et en zone semi-urbaine (50%) qu'en zone urbaine (43%). Considérée uniquement au troisième trimestre de la grossesse la carence iodée était plus accentuée en zone rurale (72%) et en zone semi-urbaine (76%) qu'en zone urbaine (57%). Inversement la surcharge en iode et l'excès d'iode chez la femme enceinte étaient plus important en zone urbaine (37% de femmes enceintes) et en zone semi-urbaine (33%) qu'en zone rurale (27%) (Tableau 2).

**Tableau 1:** Proportion des femmes enceintes (%) reparties suivant le statut iodé et l’âge de la grossesse: n = 75 femmes au premier trimestre, 75 au deuxième trimestre et 75 au troisième trimestre

Taux d'iode urinaire		Proportion des femmes enceintes (%) au			
		1^er^ trimestre	2^ème^trimestre		3^ème^trimestre	Pour les 3 trimestres
< 150 µg/l (Carence en iode)		31	56		68	52
150-249 µg/l (Statut iodé normal)		21	17		10	16
250-499 µg/l (Surcharge en iode)		38	23		14	25
≥500 µg/l (Excès d'iode)		10	4		8	7
Population étudiée (%)		100	100		100	100

nos résultats ont montré qu'au cours de la grossesse, la carence en iode était plus fréquente au troisième trimestre (68%) et au deuxième trimestre (56%) qu'au premier trimestre (31%). Moins de 20% de femmes enceintes avaient un statut iodé normal (iode urinaire entre 150 et 249 µg/l). Paradoxalement, 23% de femmes enceintes étaient confrontées à une surcharge en iode (250-499 µg/l) et 7% à un excès d'iode (>500 µg/l)

**Tableau 2:** Proportion des femmes enceintes (%) réparties suivant le milieu de recrutement: n =75 en zone rurale, 75 en zone semi-urbaine et 75 en zone urbaine

Taux d'iode urinaire		Proportion de femme (%) en zone					
		rurale	semi-urbaine		urbaine	
< 150 µg/l (Carence en iode)		62		50		43
150-249 µg/l (Statut iodé normal)		11		17		21
250-499 µg/l (Surcharge en iode)		23		22		29
≥500 µg/l (Excès d'iode)		4		11		8

L'examen du statut iodé chez les femmes suivant le milieu de recrutement a montré une plus grande proportion des femmes avec une carence iodée en zone rurale (62%) et en zone semi-urbaine (50%) qu'en zone urbaine (43%). Considérée uniquement au troisième trimestre de la grossesse, la carence iodée était plus accentuée en zone rurale (72%) et en zone semi-urbaine (76%) qu'en zone urbaine (57%). Inversement, la surcharge en iode et l'excès d'iode chez la femme enceinte étaient plus important s en zone urbaine (37% de femmes enceintes) et en zone semi-urbaine (33%) qu'en zone rurale (27%)

## Discussion

### Teneur en iode du sel de cuisine consommé à Lubumbashi

La proportion de 47,5% des sels adéquatement iodés observée dans notre travail se retrouve dans la fourchette des proportions signalées dans bon nombre d’études antérieures comme celles de Kitwa et al. [12] à Lubumbashi et d'Assoumanou et al. [13] au Benin qui rapportent respectivement 44,8 et 54,74% des sels adéquatement iodés. Mais nos résultats donnent des chiffres nettement plus élevés que ceux d'Adou et al. [6] à Abidjan ayant signalé 32% des sels adéquatement iodés. Le rapport du programme national de nutrition de la RD Congo rassemblant en 2010 des données sur 1860 ménages de la ville de Lubumbashi donnent des chiffres de loin élevés qui vont jusqu’à 66,6% des sels adéquatement iodés. Quant au sel trop riche en iode, la proportion de 7,4% observée dans notre étude est de loin inférieure à celle rapportée dans d'autres travaux comme ceux d'Assoumanou et al. [13] au Benin et d'Adou et al. [6] à Abidjan qui signalent respectivement 31,50 et 45% des sels trop riches en iode. Par ailleurs, il a été rapporté que 52,8% des sels prélevés dans les marchés au Srilanka étaient surdosés [14]. A Ndjamena, 21,4% des sels consommés étaient fortement iodés [15]. En prenant en compte la proportion des échantillons de sel adéquatement iodé et celle des échantillons de sel trop riche en iode, la disponibilité en iode du sel obtenue dans notre étude est de 54,9% et reste nettement inférieure à celle signalée dans les travaux d'Adou et al. [6] à Abidjan et d'Assoumanou et al. [13] au Benin où l'on a noté respectivement 63,5 et 99,7%. Elle est de toute manière très faible par rapport aux normes de l'OMS qui recommandent une disponibilité en iode du sel d'au moins 90% [16, 17]. Une situation plus alarmante que la nôtre a été signalée au Kyrgyzstan où Sultanalieva et al. [18] ont rapporté que 97,9% des échantillons de sel étaient iodés dont seulement 39,5% avec une teneur en iode supérieure ou égale à 15 ppm traduisant une disponibilité en iode du sel extrêmement faible. Concernant le sel faiblement iodé, il est intéressant de noter que dans notre étude, 36,9% des échantillons de sel prélevés à Lubumbashi ont une teneur en iode du sel inférieure au seuil minimal recommandé (15-40ppm). Cette proportion de sel faiblement iodé est bien supérieure à celle observée dans l’étude de Kamanda et al. [19] dont les résultats de sel faiblement iodé montrent 22,8% des échantillons de Lubumbashi et à celle trouvée à Likasi (13,9% d’échantillons récoltés) et à Kasumbalesa (34,2% d’échantillons récoltés). On constate à Lubumbashi une nette augmentation de nombre d’échantillons faiblement iodés passant de 22,8% en 2010 à 36,9% en 2012. Likasi se distingue par le nombre le plus bas d’échantillons faiblement iodés (13,9%) dans l’étude de Kamanda et al. [19]. Au vu de ces résultats, l'on note que la population de Kasumbalesa est plus exposée aux TDCI que ne le sont celle de Lubumbashi et celle de Likasi. Les sels faiblement iodés et non iodés ont aussi déjà été signalés dans d'autres villes en dehors de la RD Congo comme New Delhi où ont été observés 23% des échantillons de sels faiblement iodés et 23% de sels non iodés en dépit d'une interdiction de la vente de sels non conformes pour la consommation humaine [20]. La présence de sels non iodés ou faiblement iodés peut s'expliquer par plusieurs facteurs notamment la forme chimique d'enrichissement en iode, le mauvais conditionnement, l'exposition aux intempéries, l'introduction de sel d'exploitation artisanale non iodé et le détournement de sel destiné aux usages industriels pour l'alimentation humaine. En 2007, une étude nationale d’évaluation de la lutte contre les troubles dus à la carence en iode en République Démocratique du Congo a montré la présence dans les marchés et ménages du Katanga du sel non iodé d'exploitation artisanale en provenance des salines de Pweto et de Nguba [10, 19]. La concurrence commerciale pourrait également expliquer la présence du sel non iodé parmi les lots du sel vendus à Lubumbashi. En effet, vu que l'iodation du sel augmente le coût de production du sel de cuisine, la préférence du consommateur va bien évidemment au sel le moins cher à qualité gustative égale.

### Le statut iodé des femmes enceintes à Lubumbashi

La faible concentration d'iode urinaire chez la femme enceinte observée dans notre étude est parfaitement en accord avec les résultats obtenus à la suite de l'analyse de la teneur en iode du sel de cuisine consommé à Lubumbashi indiquant qu’à peine un échantillon de sel sur deux était adéquatement iodé. Dans les milieux carencés en iode, la femme enceinte se retrouve parmi les personnes très vulnérables [21, 22]. Il convient de noter qu'au cours de la grossesse, les besoins en iode sont considérablement augmentés [23] et qu'au sein des populations qui ne couvrent pas suffisamment leurs besoins en iode (c'est le cas de la ville de Lubumbashi), les femmes enceintes et les enfants constituent les groupes les plus vulnérables face à la carence iodée. Mais notre étude a montré que parmi les femmes enceintes, certaines d'entre elles étaient confrontées à une surcharge ou un excès d'iode et ces résultats étaient également en accord avec ceux observés sur l'analyse de la teneur en iode du sel de cuisine consommé à Lubumbashi indiquant qu'un échantillon de sel sur sept était trop riche en iode (teneur en iode du sel supérieure à 40 ppm). Enfin considérée uniquement au troisième trimestre de la grossesse, la carence iodée se révèle davantage plus préoccupante en zone rurale et en zone semi-urbaine qu'en zone urbaine. Cette situation pourrait être liée à la consommation du sel faiblement iodé ou non iodé circulant en plus grande quantité en zone rurale qu'en zone urbaine. Inversement, dans les milieux urbains circule une grande quantité de sel trop riche en iode. Ce qui explique, comme le montrent nos résultats confortés par ceux des travaux antérieurs [22], que la surcharge en iode et l'excès d'iode chez la femme enceinte soient plus accentués en zone urbaine et en zone semi-urbaine qu'en zone rurale.

## Conclusion

Le présent travail a clairement montré que la disponibilité en iode du sel consommé à Lubumbashi est faible (54.9%), se situant nettement en dessous des normes OMS (>90%). Cette situation pourrait être responsable d'une grande proportion de la carence iodée observée chez la femme enceinte (52%), ce qui expose celle-ci aux risques majeurs des troubles dus à la carence en iode.

### Etat des connaissance sur le sujet

- Il est connu qu’à Lubumbashi comme sur l'ensemble du territoire de la RD Congo: la fréquence des troubles dus à la carence iodée était extrêmement élevée avant la stratégie universelle d'iodation du sel de cuisine adoptée en 1993 à cause d'un apport insuffisant en iode alimentaire.
- La consommation du sel iodé recommandé à partir de 1994 a très sensiblement réduit les troubles dus à la carence iodée.
- La disponibilité du sel iodé évaluée dans les enquêtes de 2007 était considérablement élevée, retrouvée dans de 97,5% de ménages.

### Contribution de notre étude a la connaissance

- Notre étude apporte de nouveaux éléments sur la problématique de la consommation du sel iodé et ses conséquences à Lubumbashi en relevant les faits suivants: la disponibilité du sel iodé dans les ménages a très sensiblement diminué, étant estimée à l'issue de notre enquête à 54,9%.
- La femme enceinte se révèle la cible privilégiée de la carence iodée frappant la population de Lubumbashi.
- Face à une faible disponibilité du sel iodé dans les ménages et à une grande proportion de carence iodée observée chez la femme durant la grossesse, le couple mère-enfant est fortement menacé par les troubles dus à la carence iodée.
